# On-site communication measures as a tool in outdoor recreation management: a systematic map protocol

**DOI:** 10.1186/s13750-022-00261-3

**Published:** 2022-03-07

**Authors:** Sofie Kjendlie Selvaag, Rose Keller, Øystein Aas, Vegard Gundersen, Frode Thomassen Singsaas

**Affiliations:** 1 Norwegian Institute for Nature Research (NINA), Høgskoleringen 9, 7034 Trondheim, Norway; 2 Faculty for Environment and Natural Resource Management, Norwegian University for Life Sciences (NMBU), 1432 Ås, Norway

**Keywords:** Messaging studies, Persuasion, Human behavior, Nature-based tourism, Visitor management in national parks, Communication theory

## Abstract

**Background:**

Communication is a central tool in managing the balance between outdoor recreation and environmental protection. Several studies have evaluated different communication measures in nature area case studies, but rarely are these measures compared across contexts. We systematically map the literature guided by the question, what is the evidence base of on-site communication in outdoor recreation to change human behavior towards a more sustainable direction? Taking vulnerable natural areas as our starting point, we map distribution and abundance of communication measures, study design and outcome-related themes.

**Methods:**

The target population for our mapping review (hereafter review) are outdoor recreationists and nature-based tourists who visit natural or near-natural settings. We will examine the studies that have crafted written, oral and visual intervention measures to change behavior by using persuasion, education and information instead of legal restrictions or bans. Some examples of challenges addressed with communication measures are proper waste disposal, using designated trails, minimizing wear and tear at campsites, avoid disturbing wildlife, and encouraging appropriate and safe behavior. No geographic restrictions will be applied but we will focus on protected areas. We will search publication databases for peer-reviewed published articles using internet and specialist searches to identify grey literature in English. We will screen first by title, followed by abstract and finally full text. For each article selected for full-text screening, metadata will be extracted on key variables of interest.

The extracted data from the coding will be used to group and compare the studies to reveal knowledge gaps and knowledge clusters. We will briefly describe findings from the included studies. The review will help identify what type of human behavior researchers have addressed with communication in nature management and conservation. In addition it will highlight which communication measures are frequently used in each behavioral context. It will identify which frameworks and communication theories have been the basis for designing intervention measures and provide support to practitioners and researchers in future framing and implementation of communication measures in natural settings.

**Supplementary Information:**

The online version contains supplementary material available at 10.1186/s13750-022-00261-3.

## Background

Communication is social interaction through verbal information and nonverbal symbols where people exchange thoughts, messages or information [6, 18]. In nature-based tourism, visitor management in protected areas and outdoor recreation management in general, environmental managers have often turned to the communication process of interpretation or persuasion as a key tool in striking a balance between nature-based tourism growth and environmental protection [7, 14, 17].

There are four common principles that guide visitor management: limiting use, increasing supply in area or time used, reducing the impact of use and increasing the durability of the resource [14], p. 158). ‘Soft’ communication measures such as signage, interpretive messages and the presence of rangers can reduce the impact of use by guiding visitors towards sustainable behavior, instead of management strategies that seek to prohibit use of an area, and are in general preferred by visitors, managers and decision-makers [14], p. 41). Though these principles are widely accepted in North American contexts, internationally there remains much debate on appropriate measures in protected areas and very little research has been done on the effectiveness of different types [11], p. 38).

There is an almost endless menu of communication measures that have been implemented in natural settings used for recreation and, while their effectiveness is subject to many situational aspects, studies exist that have evaluated their effectiveness in outdoor recreation [4, 20–22, 25]. However, the measures and contexts are to a lesser extent seen in relation to each other and a preliminary search did not identify any existing cross-comparative reviews. Furthermore, existing literature addresses only singular aspects of communication theory, or examines outcomes in visitor satisfaction, knowledge gains and attitudes rather than behavioral change [3, 16, 23, 26]. Munro et al. [16] reviewed a sample of the literature on communication in natural areas, but the methods used in the reviewed studies are insufficiently described and cannot openly be replicated, and only two of the studies were identified measuring behavioral change. Kidd et.al [10] reviewed and investigated conservation messaging research but, similarly, most studies looked at awareness or only encouraged behavioral change. The review only looked at communication related to conservation and did not examine the studies’ measures, design, or context. The authors suggest further research to facilitate development of a better understanding of the influence of communication on visitor behavior [10, 16]. We suggest that a systematic map in this context has the potential to bring together information about visitor behavior and improve the knowledge of how behavioral change towards more sustainable practices has been researched through soft communication measures. An overview of evidence pertaining to communication measures is also highly sought after by environmental practitioners in general, as well as a natural point of departure for novel research on the topic. In Norway, this work is especially sought after by our local stakeholders, including environmental managers, tourism industry leaders, farming and timber producers, fishing, hunting and hiking association members. A recent series of workshops with these stakeholders clearly stated the need for more experience and knowledge on various measures to strike a balance between natural values and visitor experience values in outdoor recreation. Our research question is guided by the discourse in these workshops and we will report the findings back to our stakeholders although they will not further influence or take part in the review.

Human behavior in nature is heavily context dependent and it is important to be aware that a communication measure that works one place and at one time may not work another place or at another time [1, 2, 9, 13, 24]. Many factors influence behavior, therefore the same measure might affect behavior differently among people with different backgrounds. Also, the same measure might work differently within one person, e.g. different trip-mode like daytrip with family or wilderness adventure with friends, and during different phases of life. Yet, due to established frameworks for understanding and analyzing human behavior it is likely that a review can identify some general findings about the settings and management guidelines that can help in the design of future communication measures. This review will also assist in identifying what common challenges in visitor management are addressed with communication measures. It will highlight which theories have been the basis for designing communication measures and help support future visitor behavior strategies in outdoor recreation. The review will also identify knowledge gaps as well as challenges communication-behavior studies often face.

The study will only map clearly defined on-site communication measures or measures in experimental settings that aim to influence behavior at that specific time and exclude communication given before visitation using internet and printed matter such as newspapers, books etc. Our review will have a specific focus on protected areas and not address behavior where people knowingly have engaged in illegal behavior or vandalism.

## Objective of the review

### Primary question

-What is the evidence base of on-site communication in outdoor recreation to change human behavior towards a more sustainable direction?
- P: people participating in outdoor recreation,
- I: on-site communication measures (in situ),
- C: no communication measures,
- O: changed behavior.

### Secondary questions

-Which theories and conceptual frameworks have been used to guide empirical studies on the effects of communication in guiding human behavior in natural settings?
-What types of research design and methods, e.g. along the measurement and representational dimensions, have been used to evaluate the effectiveness of on-site communication measures in natural settings?
-What type of unsustainable or unsafe human behavior has been handled with on-site communication in visitor management in natural settings?
-Which on-site communication measures have been studied in the context of visitor management?

## Methods

### Searching for articles

Systematic searches will be performed using English search terms in the databases Scopus and the following databases from Web of Science Core Collection:

Science Citation Index Expanded (SCI-EXPANDED): 1987–present.

Social Sciences Citation Index (SSCI): 1987–present.

Arts and Humanities Citation Index (AHCI): 1987–present.

Emerging Sources Citation Index (ESCI): 2015–present.

Several preliminary search terms were tested and combined in Web of Science to find a best possible search string (see Appendix section for details on test searches). The comprehensiveness of the search string has been tested by assessing whether articles of known relevance were returned by searches in the selected bibliographic databases. We identified the benchmark articles using our own experience in the field; these articles are listed in Appendix section. For any article not retrieved by our benchmark search, we identified which concept category was not being matched and which relevant terms from the title, abstract, and keywords of missing articles should be added to our search string. Search terms describing the population (people participating in outdoor recreation in natural settings) were combined with terms describing intervention [on-site communication measures (in situ)] and terms describing outcome (changed behavior). The search terms were cleaned up to avoid redundant terms and added the asterisk symbol (*) to capture plurals. The updated search string returned 4352 papers (December 16th 2021) and included all six benchmark articles:

(TS = ("nature-based tourism" OR "nature area*" OR "protected area*" OR forest* OR "open space*" OR park* OR beach* OR backcountry OR "recreation" OR wilderness OR mountain*)).

AND

(TS = (Communicat* OR messag* OR info* OR learn* OR persua* OR interpret* OR educat*)).

AND

TS = (((change* OR influenc* OR impact* OR guid* OR regulat* OR modify OR Effect*) NEAR/5 behavio*) OR ((change* OR influenc* OR impact* OR guid* OR regulat* OR modify OR Effect*) NEAR/5 experience*) OR ((change* OR influenc* OR impact* OR guid* OR regulat* OR modify OR Effect*) NEAR/5 safe*) OR ((change* OR influenc* OR impact* OR guid* OR regulat* OR modify OR Effect*) NEAR/5 pay*) OR ((change* OR influenc* OR impact* OR guid* OR regulat* OR modify OR Effect*) NEAR/5 responsibility) OR ((change* OR influenc* OR impact* OR guid* OR regulat* OR modify OR Effect*) NEAR/5 "visitor education")).

The English string detailed above resulted from our benchmark search. We will use this string in WoS and a similar string will be used in Scopus, adjusted to the interface. To ensure that we obtain all relevant literature we will conduct forwards and backwards citation searching. Here we will use the benchmark articles as reference points and then review the literature backward and forward in time. This approach can be viewed as a type of snowball or chain sampling [15], used in this case in conjunction with written sources and not with informants. We will search for grey literature studies adapting the same search strings via Google Scholar and reaching out to stakeholder contacts to find theses, reports and articles not indexed in the search databased used. As substantial work in visitor behavior and communication measures have been conducted in the U.S., we will also use the US Dept. of Interior Integrated Resource Management database (IRMA) for studies conducted within US public/protected lands. A flow chart in the final report will illustrate all identified studies, the excluded studies at each stage with reasons for exclusion at full text [5]. If applicable, a list of unobtainable articles will be provided in Additional file 1.

### Article screening and study eligibility criteria

#### Screening process

All publication database results will be downloaded to Endnote and its auto-deduplication process will be used. The results will then be uploaded to a public project on SysRev.com for screening. Full text screening is proceeded by title and abstract level assessment. We will address intercoder reliability providing all coders the same 25 studies to screen at the abstract level. Disagreements will be discussed, and the eligibility criteria can be refined to give the coders a more equal understanding of the criteria set for this review. The remaining articles for title and abstract level assessment will be divided equally between all four reviewers and screened independently. To ensure that there is consistently applied the coders will then screen 25 new studies in duplicate at full text level and all screened studies in duplicate will be resolved by consensus or by one of the experienced authors. Two reviewers will proceed by screening different full text articles, code them and check for consistency when uncertainty in codes arise. Replicability of eligibility decisions will be measured and reported and the excluded articles at full text screening level (and reasons for this) will be provided as an additional file in the final review report. Results of searching and screening will be reported following ROSES guidance [5].

### Eligibility criteria

Records will be included in this review if they meet the following criteria.

#### Population

People participating in outdoor recreation including hiking, skiing, guided tours, sport activities, cycling, horseback riding, dog sledding, hunting and/or fishing, mushroom/berry/plant picking, photographing and picnicking as long the activities take place in outdoor areas which are not heavy facilitated, as sporting arenas or within commercial skiing resorts. Examples of natural areas: forests, mountain areas, bushlands and lakes/rivers. Study areas might include any geographic region globally.

#### Intervention

Any implemented communication measure, both written/printed, visual and oral/audio based on information, learning, persuasion and interpretation where the wanted outcome is to encourage pro-environmental behavior. In our review, pro-environmental behavior includes proper waste disposal, staying on designated trails, minimizing campfire impacts, respecting and not feeding wildlife, fee and regulation compliance, showing consideration towards other visitors and following safety measures. Measures seeking to change behavior where it is clear that people knowingly have engaged in illegal behavior or vandalism will be excluded.

#### Comparator

No communication measure at the same place, but at a different time or in a similar setting or testing the effect of different communication measures at the same place or in similar settings.

#### Outcome

Changed behavior including both wanted and unwanted behavior based on how it is affecting the environment or people. Because outcomes are typically reported in studies as degrees of impact to behavior and/or observed impacts on the environment we expect primarily quantitative data. Changed behavior should be linked to the intervention, but the methods used and how changed behavior is reported can differ. We will also include qualitative data because it provides vital context for the design of communication measures and suite of behaviors in a given natural setting.

#### Study type

Any primary empirical research study, both observational studies and experimental/intervention studies published as reports or articles. Both quantitative and qualitative data will be included. We anticipate mostly quantitative data, but include recognized methods of qualitative data collection (e.g. interviews, focus groups) and analysis (e.g. thematic analysis, grounded theory). We will exclude books, reviews, model studies and meta-analyses.

We will ensure that no reviewer screens records for any article that they have authored themselves.

### Study validity assessment

Since our aim is to map the challenges in outdoor recreation that have been addressed through communication measures, we do not intend to conduct an appraisal of the validity of included studies. However, we acknowledge that validity, in the sense of correspondence between construct and measurement, is a common challenge for studies including how people interpret and evaluate different stimuli in a communication process. Our review will provide some preliminary estimate of the quality of the available evidence by briefly describing the design of each study.

### Data coding strategy

All studies that pass the eligibility criteria will be included in the data coding and mapping. The four reviewers will extract data from the same 10 full text articles independently. Based on this, differences in extraction of meta data and coding will be identified, discussed and resolved. Then a single reviewer will extract data from the remaining studies at full text level. The meta data and themes considered in our review are listed in the Table 1. Items can be reduced based on the number of studies included in our review and available resources. The list was compiled using *Communication research in outdoor recreation and natural resources management* [2], *Influencing human behavior* [13], *Navigating Environmental Attitudes* [9], *Promoting persuasion in protected areas* [8] and *Social Science Theory for Environmental Sustainability* [24]. These books and reports guided and helped identify relevant themes for our review. The list was also vetted according to input by external experts. Where needed, we will contact the authors to request missing data records in line with the items listed in Table 1. We will also make sure that no reviewer extracts data from their own work. The extracted data records will be made available in an excel spreadsheet as an additional file in the final review.

**Table 1:** Description of metadata and themes to be coded and considered in the systematic map

Item	Description
Title	Published title
Year	Year of publication
Date	Date of publication
Jounal	Journal name
DOI	Identification code if available
Authors	Author’s records
Abstract	Published summary text
Behavior description(s)	Authors description of the behavior being targeted:
Behavioral category (Ham et al. [8], p 10. Roggenbuck [19], p 150–162)	Give the study a category based on the wanted change in behavior (multiple categories can be applied to a single study, but only if it is mentioned by the authors) 1 = Disposing waste properly (e.g. ‘pack in pack out’ or dispose waste in trash cans) 2 = Channeling use (e.g. hiking on designated trails) 3 = Minimizing camping impacts (e.g. not damaging trees or use of designated camping and/or campfire areas) 4 = Respect wildlife (e.g. do not feed or disturb wildlife or maintain safe distance) 5 = Pay fees and comply with regulations (e.g. no collecting or harvesting of what and where it is not allowed, paying fishing license or paying for public goods such as toilets and shelters or paying a national park fee) 6 = Social impacts (e.g. show consideration to other visitors) 7 = Act according to safety measures (e.g. wear proper clothing or staying away from a place because of safety reasons) 8 = Other behavior
Study content	Give the study a category based on setting: 1 = Experiment (experimental setting) 2 = Quasi-experiment (experimental, but more adapted to real life conditions) 3 = Observation (real life setting)
Study experimental design	Give the study a category based on study design. Several can apply: 1 = Pre- and post-test used 2 = Using a control group
Study measurement	Give the study a category based on how the behavioral change was measured (multiple categories can be applied to a single study) 1 = Observation of behavior 2 = Counter 3 = Big data 4 = GPS 5 = Interviews 6 = Focus groups 7 = Thematic qualitative 8 = Intercept survey 9 = Passive survey (e.g. online) 10 = Survey including qualitative components 11 = Assessments of impacts on nature (field survey) 12 = Other methods, explain:
Study design comments	Describe the study design in more detail and what the methods described separately:
Study period	State number of days the behavior was studied
Sample size	Number of respondents—State number of people who got their behavior studied
Targeted population	Stated population that is the focus of the behavior(s) (age, gender, local–regional-national, urban–rural etc.)
Population category	Give the study a category based on focus population: 1 = Mostly locals 2 = Mostly residents within the country 3 = Mostly foreigners 4 = Mix of different visitors 5 = Not stated
Country	State the country in which the study takes place:
Geographical scope	Give the study a category for in what region it takes place: 1 = Europe 2 = US and Canada 3 = Latin America 4 = Asia 5 = Africa 6 = Oceania 7 = Not stated
Environment setting category	Give the study a category for what setting/ecosystem it takes place: 1 = Forest 2 = Woodland-grassland (Bushland/savannah) 3 = Mountain 4 = Beach 5 = Freshwater (On Lake/river) 6 = Marine (Near/ on the ocean, coral reefs etc.) 7 = Park (open place in a city or town) 8 = Desert 9 = Other setting, please specify: 10 = Not stated
Protected areas	Give the study a category if the study has taken place in a protected area: 2 = Taken place in a protected area 1 = Not taken place in a protected area, but similar setting and no big reasons for the results to change drastic if it was conducted in a protected area 0 = Different setting, behavior and/or population than what can appear in protected areas
Protected area category	If the category above was rated 2 categorize protection according to IUCN: 1a = Strict Nature Reserve 1b = Wilderness Area 2 = National Park 3 = Natural Monument or Feature 4 = Habitat/Species Management Area 5 = Protected Landscape/Seascape 6 = Protected area with sustainable use of natural resources 7 = Not able to categorize
Theory/framework	State the different communication theories/frameworks that have been used to develop the communication measure. If it cannot be found write 0:
Theory/framework category (Stern 2018, see Table 3.1 p. 22–25 for theories under persuasive communication, p 27–70, p 84–120)	If not given 0 above, give the study a category for what theory/framework it has focused on (several categories can be given if applied): 1 = Norm theory (social norm, norm activation and value-belief-norm) 2 = Cognitive dissonance 3 = Elaboration Likelihood Model 4 = Theory of planned behavior 5 = Self-Determination Theory 6 = Extended Parallel Process Model of Fear Appeals 7 = Motivation Crowding Theory 8 = Maslow’s Hierarchy of Needs 9 = Identity Theory 10 = Moral Foundations Theory 11 = Frame theory 12 = Meyer’s Culture Map 13 = Trust Theory 14 = Principled Negotiation 15 = Diffusion Theory 16 = Other
Communication measures category	Give the study a category based on how the message was communicated: 1 = Written 2 = Oral
Medium written category	If the category was rated 1 above give the study a category based on how the message was communicated (can be extended): 1 = Sign 2 = Poster (longer explanation than sign) 3 = Brochure 4 = Digital display 5 = Multimedia/infotainment
Medium oral category	If the category was rated 2 above, give the study a category based on how the message was communicated (can be extended): 1 = Person 2 = Soundtrack 3 = multimedia/infotainment
Message focus category Categories based on persuasive communication theories in Stern 2018, p. 27–70, p. 84–120 and topics focused on in Absher and Bright 2004, p. 117–126 and Heberlein 2012, structural fixes (norms p. 90–112) and cognitive fixes (attitudes, direct experience, identity p. 15–68)	Give the study one or more categories based on what the message is focusing on: 1 = Feelings/emotions (e.g. pride, fear, appreciation, responsibility) 2 = Education/knowledge (e.g. reliable evidence, certainty, give a solution/outcome, consequence, competence, remove barriers) 3 = Activating existing knowledge/experience (e.g. confirmatory thought, direct experience, prior knowledge) 4 = Feed-back (sign pledge, build personal relationships etc.) 5 = Identity (relatedness/relevance/meaningfulness, autonomy, freedom, not shamed about prior experience, important reference group/role models, cultural cognition) 6 = Social (e.g. relationship between people, status is social group, collective, pride, shame) 7 = Environment (sustainable development, climate, biodiversity etc.) 8 = Personal/local/ place-based message: connection between visitor and the site/resource, two-way dialogue 9 = Acknowledgement/reward/benefits 10 = Punishment/sanction/cost of action 11 = Experts/management (source of the message or backing it up, credibility, respect, trust) 13 = Provocation (personally reflection on content and its deeper meanings) 14 = Other, please explain:
Confounding variables identified category	State if data was not in accordance with expectations: 1 = Yes (confounding variable) 2 = No
Confounding variables description	If the category was rated 1, specify which one
Reasons for unaltered behavior	Describe authors’ reasoning for observed unaltered behavior despite tested measures (e.g. context, if you do not have litter to toss), identity, cognitive dissonance, self-justification, undermining credibility, ability to process the message etc.)

### Study mapping and presentation

The final report published in Environmental Evidence will include the study mapping and presentation. The presentation of the collected studies and the data they contain rely primarily on the extracted data records (see Table 1). The extracted data consists of text and coding as the map is focusing on a wide range of questions. The presentation of data will also be based on grouping and clustering. The studies will be organized by behavior category, context, targeted population, study design and outcomes. A figure will illustrate how the relevant literature is organized and descriptive statistics regarding relevant information on the distribution of the articles will be provided in the report. We will explain how our review can be used to find appropriate studies and observations on the distribution of articles. We will use clusters to explore relationships within and between studies. This will allow identification of key knowledge gaps, knowledge clusters and locate characteristics that can help explain the effectiveness of on-site communication measures to change human behavior in outdoor recreation.

The mapping methods and focus will depend on the diversity in methodology and the number of studies included in the review. The findings will be presented visually in the form of histograms. The coding dataset will also be used to map what type of challenges that have been addressed through communication measures and will be graphically displayed as bubble charts. We will identify contexts where there are few studies (e.g. geographic scope) and clear gaps (e.g. targeted behavior and population) guided by our coding dataset where communication theory in outdoor recreation can be more developed. We will use the visualizations we produce to identify underrepresented types of behavior, settings, and regions in the evidence base and highlight where more research should be conducted. The data will consist of case studies which will be used to summarize the state of evidence base in terms of their distribution, abundance and trends in relation to the secondary questions and highlight theoretical principles and concepts in communication design [27]. We will include quantitative (e.g. collected from surveys, tracking-data) and qualitative data (e.g. collected from interviews) focused studies which may lead to a more holistic and comprehensive map of different perspectives on visitor behavior in the context of outdoor recreation management.

We will only include literature written in English due to available resources. Thus, a substantial part of the evidence base will not be assessed. The impact of this on the mapping outcomes is uncertain but we expect some degree of geographical skewness. Statistically significant results (positive results) are more likely to be published than non-significant ones (negative results) and this can lead to overstating the effect of communication interventions [12]. These asymmetries and potential biases will be acknowledged in the report. We will try to minimize bias in the search for articles by looking for evidence outside traditional academic electronic bibliographic sources, use multiple databases and include searches for older publications and grey literature [12].

## Supplementary Information

**Additional file 1:** ROSES form for Systematic Map Protocols.

## Data Availability

Data analyzed during this study is included in this published article and an overview and reference list of all data used are available in this article’s supplementary information files. The results from the systematic searches and article screening are also available in Sysrev (sofie.selvaag/ On-site communication measures as a tool in outdoor recreation management).

## First test search

We ran search tests in Web of Science on September 23rd 2021. This was the first search string:

(TS = ("Outdoor recreation" OR "national park*" OR "nature-based tourism" OR "nature area*" OR "protected area*" OR forest OR "open space" OR park OR beach OR backcountry OR "recreation area*" OR wilderness OR mountain)).

AND

(TS = (Communicat* OR messag* OR info* OR learn* OR persua* OR interpret* OR educat*)).

AND

TS = (Steer* OR Change* OR Alter* OR influenc* OR effect* OR impact* OR control* OR affect* OR guid* OR regulat* OR modify* OR influenc* OR (reduc* AND behavio*) OR practice* OR action* OR waste OR litter* OR camp* OR impact* OR wildlife impact* OR social OR safety OR pay* OR trail OR rules OR regulat*).

The result of the first test search was 92,998 papers in Web of Science alone – a number too high for screening. Especially the Outcome-terms produced a large number of publications – more than 25 million.

## Second test search

For test search 2 the idea was to use the Boolean operator AND between the change-terms and the behavior-terms in the outcome-section, to narrow down the results. This was the search string:

(TS = ("Outdoor recreation" OR "national park*" OR "nature-based tourism" OR "nature area*" OR "protected area*" OR forest OR "open space" OR park OR beach OR backcountry OR "recreation" OR wilderness OR mountain)).

AND

(TS = (Communicat* OR messag* OR info* OR learn* OR persua* OR interpret* OR educat*)).

AND

TS = ((change* OR influenc* OR impact* OR guid* OR regulat*OR modify OR Effect* OR Affect*) AND (behavio* OR experience* OR safe*OR pay*)).

The result from test search 2 was 12,804 articles. A significantly lower number – but still a bit high, especially since this will increase quite a bit when the results from SCOPUS search will be added.

## Third test search

The Boolean operator AND was replaced by the proximity operator “NEAR/5”, in attempt to narrow the result list down further – hopefully without losing relevant publications. The following search string gave a result of 3456 papers:

(TS = ("Outdoor recreation" OR "national park*" OR "nature-based tourism" OR "nature area*" OR "protected area*" OR forest OR "open space" OR park OR beach OR backcountry OR "recreation" OR wilderness OR mountain)).

AND

(TS = (Communicat* OR messag* OR info* OR learn* OR persua* OR interpret* OR educat*)).

AND

TS = (((change* OR influenc* OR impact* OR guid* OR regulat* OR modify OR Effect* OR Affect*) NEAR/5 behavio*) OR ((change* OR influenc* OR impact* OR guid* OR regulat* OR modify OR Effect* OR Affect*) NEAR/5 experience*) OR ((change* OR influenc* OR impact* OR guid* OR regulat* OR modify OR Effect* OR Affect*) NEAR/5 safe*) OR ((change* OR influenc* OR impact* OR guid* OR regulat* OR modify OR Effect* OR Affect*) NEAR/5 pay*)).

At this point the following pre-made benchmark list of 13 articles was involved:

- 1. Bradford LE, McIntyre N (2007) Off The Beaten Track: Messages As A Means Of Reducing Social Trail Use At St. Lawrence Islands National Park. Journal of Park & Recreation Administration 25 (1)
- 2. Brown PJ, Hunt JD (1969) The influence of information signs on visitor distribution and use. Journal of Leisure Research 1 (1):79-83
- 3. Brown TJ, Ham SH, Hughes M (2010) Picking up litter: an application of theory-based communication to influence tourist behaviour in protected areas. Journal of Sustainable Tourism 18 (7):879-900. https://doi.org/10.1080/09669581003721281
- 4. Freuler B, Hunziker M (2007) Recreation activities in protected areas: bridging the gap between the attitudes and behaviour of snowshoe walkers. Forest Snow and Landscape Research 81 (1/2):191-206
- 5. Gramann JH, Bonifield RL, Kim YG (1995) Effect of personality and situational factors on intentions to obey rules in outdoor recreation areas. Journal of Leisure Research 27 (4):326-343. https://doi.org/10.1080/00222216.1995.11949753
- 6. Krumpe EE, Brown PJ (1982) Redistributing backcountry use through information related to recreation experiences. Journal of Forestry 80 (6):360-364. https://doi.org/10.1093/jof/80.6.360
- 7. Oliver SS, Roggenbuck JW, Watson AE (1985) Education to reduce impacts in forest campgrounds. Journal of Forestry 83 (4):234-236. https://doi.org/10.1093/jof/83.4.234
- 8. Roggenbuck JW, Berrier DL (1982) A comparison of the effectiveness of two communication strategies in dispersing wilderness campers. Journal of Leisure Research 14 (1):77-89. https://doi.org/10.1080/00222216.1982.11969506
- 9. Saunders R, Weiler B, Scherrer P, Zeppel H (2019) Best practice principles for communicating safety messages in national parks. Journal of Outdoor Recreation and Tourism-Research Planning and Management 25:132-142. https://doi.org/10.1016/j.jort.2018.01.006
- 10. Schwartz F, Taff BD, Lawhon B, VanderWoude D (2018) Mitigating Undesignated Trail Use: The Efficacy of Messaging and Direct Site Management Actions in an Urban-Proximate Open Space Context. Environmental Management 62 (3):458-473. https://doi.org/10.1007/s00267-018-1054-1
- 11. Settina N, Marion JL, Schwartz F (2020) Leave No Trace Communication: Effectiveness Based on Assessments of Resource Conditions. Journal of Interpretation Research 25 (1):5-25. https://doi.org/10.1177/1092587220963523
- 12. Steckenreuter A, Wolf ID (2013) How to use persuasive communication to encourage visitors to pay park user fees. Tourism Management 37:58-70. https://doi.org/10.1016/j.tourman.2013.01.010

- 13. Taff D, Newman P, Lawson SR, Bright A, Marin L, Gibson A, Archie T (2014) The role of messaging on acceptability of military aircraft sounds in Sequoia National Park. Applied Acoustics 84:122-128. https://doi.org/10.1016/j.apacoust.2013.09.012

Four of the papers above (#2, #6, #7 and #8) are too old to be indexed in WoS. Three of the remaining nine (#1, #4 and #11) are published in journals not indexed in WoS. That left six articles (#3, #5, #9, #10, #12 and #13) which are indexed in WoS. Out of the six, four papers (#3, #9, #12 and #13) were among the 10,655 papers in the third test search. #5 and #10 were missing.

## Fourth test search

Further investigation of the two missing papers led to the inclusion of two new terms in the outcome section: responsibility and "visitor education". The updated search string was like this:

(TS = ("Outdoor recreation" OR "national park*" OR "nature-based tourism" OR "nature area*" OR "protected area*" OR forest OR "open space" OR park OR beach OR backcountry OR "recreation" OR wilderness OR mountain)).

AND

(TS = (Communicat* OR messag* OR info* OR learn* OR persua* OR interpret* OR educat*)).

AND

TS = (((change* OR influenc* OR impact* OR guid* OR regulat* OR modify OR Effect* OR Affect*) NEAR/5 behavio*) OR ((change* OR influenc* OR impact* OR guid* OR regulat* OR modify OR Effect* OR Affect*) NEAR/5 experience*) OR ((change* OR influenc* OR impact* OR guid* OR regulat* OR modify OR Effect* OR Affect*) NEAR/5 safe*) OR ((change* OR influenc* OR impact* OR guid* OR regulat* OR modify OR Effect* OR Affect*) NEAR/5 pay*) OR ((change* OR influenc* OR impact* OR guid* OR regulat* OR modify OR Effect* OR Affect*) NEAR/5 responsibility) OR ((change* OR influenc* OR impact* OR guid* OR regulat* OR modify OR Effect* OR Affect*) NEAR/5 "visitor education")).

The result was 3512 papers in WoS. Now, all six benchmark-articles were included.

## Fifth test search

The term “affect*” was removed from the outcome section, since this term led to quite a few papers on nature therapy. The updated search string gave a result of 3135 papers:

(TS = ("Outdoor recreation" OR "national park*" OR "nature-based tourism" OR "nature area*" OR "protected area*" OR forest OR "open space" OR park OR beach OR backcountry OR "recreation" OR wilderness OR mountain)).

AND

(TS = (Communicat* OR messag* OR info* OR learn* OR persua* OR interpret* OR educat*)).

AND

TS = (((change* OR influenc* OR impact* OR guid* OR regulat* OR modify OR Effect*) NEAR/5 behavio*) OR ((change* OR influenc* OR impact* OR guid* OR regulat* OR modify OR Effect*) NEAR/5 experience*) OR ((change* OR influenc* OR impact* OR guid* OR regulat* OR modify OR Effect*) NEAR/5 safe*) OR ((change* OR influenc* OR impact* OR guid* OR regulat* OR modify OR Effect*) NEAR/5 pay*) OR ((change* OR influenc* OR impact* OR guid* OR regulat* OR modify OR Effect*) NEAR/5 responsibility) OR ((change* OR influenc* OR impact* OR guid* OR regulat* OR modify OR Effect*) NEAR/5 "visitor education")).

The six benchmark-articles were then checked against the new result list, and all six were still included.

## References

[1] Absher, J. Parables and paradigms: an introduction to using communication theories in outdoor recreation research.1998.

[2] Absher JD, Bright AD. Communication research in outdoor recreation and natural resources management. Society and Natural Resources—a summary of knowledge: Jefferson, Missouri, Modern Litho. 2004:117–26.

[3] Ardoin NM, Wheaton M, Bowers AW, Hunt CA, Durham WH. Nature-based tourism’s impact on environmental knowledge, attitudes, and behavior: a review and analysis of the literature and potential future research. J Sustain Tour. 2015;23(6):838–58. 10.1080/09669582.2015.1024258

[4] Brown TJ, Ham SH, Hughes M. Picking up litter: an application of theory-based communication to influence tourist behaviour in protected areas. J Sustain Tour. 2010;18(7):879–900. 10.1080/09669581003721281

[5] Haddaway NR, Macura B, Whaley P, Pullin AS. 2017. ROSES for systematic map protocols. Version 1.0. 10.6084/m9.figshare.5897284.

[6] Hansen A. Promising directions for environmental communication research. Environ Commun. 2015;9(3):384–91. 10.1080/17524032.2015.1044047

[7] Ham S. Interpretation: making a difference on purpose. Golden: Fulcrum publishing; 2016.

[8] Ham S, Brown T, Curtis J, Weiler B, Hughes M, Poll M. Promoting persuasion in protected areas: a guide for managers who want to use strategic communication to influence visitor behaviour. Technical report. 2009.

[9] Heberlein, Thomas A. Navigating environmental attitudes. 2012: 583–585. 10.1111/j.1523-1739.2012.01892.x 22809349

[10] Kidd LR, Garrard GE, Bekessy SA, Mills M, Camilleri AR, Fidler F, Fielding KS, Gordon A, Gregg EA, Kusmanoff AM, Louis W. Messaging matters: a systematic review of the conservation messaging literature. Biol Cons. 2019;1(236):92–9. 10.1016/j.biocon.2019.05.020

[11] Leung YF, Spenceley A, Hvenegaard G, Buckley R. Tourism and visitor management in protected areas: guidelines for sustainability, vol. 27. Gland: IUCN; 2018.

[12] Livoreil B, Glanville J, Haddaway NR, et al. Systematic searching for environmental evidence using multiple tools and sources. Environ Evid. 2017;6:23. 10.1186/s13750-017-0099-6. 10.1186/s13750-017-0099-6

[13] Manfredo MJ. Influencing human behavior: theory and applications in recreation, tourism, and natural resources management. Champaign: Sagamore Pub Llc; 1992.

[14] Manning RE, Anderson LE, Pettengill P. Managing outdoor recreation: case studies in the national parks. Wallingford: CABI; 2017.

[15] Miles MB, Huberman AM. Qualitative data analysis: an expanded sourcebook. Thousand Oaks: SAGE; 1994.

[16] Munro JK, Morrison-Saunders A, Hughes M. Environmental interpretation evaluation in natural areas. J Ecotour. 2008;7(1):1–4. 10.2167/joe137.0

[17] Newsome D, Moore SA, Dowling RK. Natural area tourism: ecology, impacts and management. Bristol: Multilingual Matters; 2012.

[18] Pezzullo PC, Cox R. Environmental communication and the public sphere. Thousand Oaks: Sage Publications; 2017.

[19] Roggenbuck JW. Use of persuasion to reduce resource impacts and visitor conflicts. Influ Hum Behav. 1992:149–208.

[20] Saunders R, Weiler B, Scherrer P, Zeppel H. Best practice principles for communicating safety messages in national parks. J Outdoor Recreat Tour. 2019;1(25):132–42. 10.1016/j.jort.2018.01.006

[21] Schwartz F, Taff BD, Lawhon B, VanderWoude D. Mitigating undesignated trail use: the efficacy of messaging and direct site management actions in an urban-proximate open space context. Environ Manag. 2018;62(3):458–73. 10.1007/s00267-018-1054-1 29740681

[22] Steckenreuter A, Wolf ID. How to use persuasive communication to encourage visitors to pay park user fees. Tour Manag. 2013;1(37):58–70. 10.1016/j.tourman.2013.01.010

[23] Stern MJ, Powell RB. What leads to better visitor outcomes in live interpretation? J Interpret Res. 2013;18(2):9–43. 10.1177/109258721301800202

[24] Stern MJ. Social science theory for environmental sustainability: a practical guide. Oxford: Oxford University Press; 2018.

[25] Taff D, Newman P, Lawson SR, Bright A, Marin L, Gibson A, Archie T. The role of messaging on acceptability of military aircraft sounds in Sequoia National Park. Appl Acoust. 2014;1(84):122–8. 10.1016/j.apacoust.2013.09.012

[26] Wearing SL, Whenman AE. Tourism as an interpretive and mediating influence: a review of the authority of guidebooks in protected areas. Tour Anal. 2009;14(5):701–16. 10.3727/108354209X12597959733936

[27] Yin RK. Case study research and applications design and methods. 6th ed. Thousand Oaks: SAGE; 2018.

