# Supplementary material for: On-site communication measures as a tool in outdoor recreation management: a systematic map protocol
Source: Environ Evid. 2022 Mar 7;11:7. doi: 10.1186/s13750-022-00261-3 (PMC11378849; doi:10.1186/s13750-022-00261-3)
Supplement: Supplementary file 1 — Additional file 1: ROSES form for Systematic Map Protocols. [file 13750_2022_261_MOESM1_ESM.pdf]

Section / sub-section	Topic	Description	Further explanation	Checklist/Meta-data	Author response	Comments
Title	Title	The title must indicate that it is a systematic map protocol, and must indicate if it is an update/amendment: e.g. "A systematic map update protocol...".	The title should normally be the same or very similar to the review question.	Meta-data	On-site communication measures as a tool in outdoor recreation management: a systematic map protocol	
Type of review	Type of review	Select one of the following types of review: systematic map, systematic	See CEE Guidance on systematic mapping [1], and on amendments and	Meta-data	systematic map	
Authors contacts	Authors contacts	The full names, institutional addresses, and email addresses for all		Checklist	Yes	
Abstract	Structured summary	Abstract must not exceed 350 words and must include two sections 1)		Checklist	Yes	
Background	Background	Describe the rationale for the review in the context of what is already	A theory of change and/or conceptual model can be presented that links	Checklist	Yes	
Stakeholder engagement	Stakeholder engagement	The planned/actual role of stakeholders throughout the review process		Checklist	Yes	
Objective of the review	Objective	applicable).	questions are usually linked to sources of heterogeneity (effect	Checklist	Yes	
	Definitions of the question components	Break down and summarise question key elements e.g. population, intervention(s)/exposure(s), comparator(s), and outcome(s).	For other question types see [4,5]	Meta-data	P: people participating in outdoor recreation<br>I: on-site communication measures (in situ)<br>C: no communication measures<br>O: changed behavior	Primary question: What is the evidence base of on-site communication in outdoor recreation to change human behavior towards a more sustainable direction?
Methods						
Searches	Search strategy		Details regarding search strategy testing should be provided.	Checklist	Yes	
	Search string	Provide Boolean-style full search string and state the platform for which the string is formatted (e.g. Web of Science format)		Meta-data	Scopus and Web of Science: ("nature-based tourism" OR "nature area*" OR "protected area*" OR forest* OR "open space*" OR park* OR beach* OR backcountry OR "recreation" OR wilderness OR mountain*)<br>AND<br>(Communicat* OR message* OR info* OR learn* OR persua* OR interpret* OR educat*)<br>AND<br>(((change* OR influenc* OR impact* OR guid* OR regulat* OR modify OR Effect*) NEAR/5 behavio*) OR ((change* OR influenc* OR impact* OR guid* OR regulat* OR modify OR Effect*) NEAR/5 experience*) OR ((change* OR influenc* OR impact* OR guid* OR regulat* OR modify OR Effect*) NEAR/5 safe*) OR ((change* OR influenc* OR impact* OR guid* OR regulat* OR modify OR Effect*) NEAR/5 pay*) OR ((change* OR influenc* OR impact* OR guid* OR regulat* OR modify OR Effect*) NEAR/5 responsibility) OR ((change* OR influenc* OR impact* OR guid* OR regulat* OR modify OR Effect*) NEAR/5 "visitor education"))	
	Languages – bibliographic	List languages to be used in bibliographic database searches.		Meta-data	English	
	Languages – grey literature	List languages to be used in organizational websites searches and web-		Meta-data	English	
	Bibliographic databases	Provide the number of bibliographic databases to be searched.		Meta-data	2	
	Web – based search engines	Provide the number of web – based search engines to be searched.		Meta-data	1	
	Organisational websites	Provide the number of organisational websites to be searched.		Meta-data	1	
	Estimating the	Describe the process by which the comprehensiveness of the search		Checklist	Yes	
	Search update	Describe any plans to update the searches during the conduct of the	Optional. A search update is good practice if original searches were	Checklist	n/a	
Article screening and study	Screening strategy	Describe the methodology for screening articles/studies for		Checklist	Yes	
	Consistency checking	Describe clearly the process for checking consistency of decisions		Checklist	Yes	
	Inclusion criteria	Describe the inclusion criteria used to assess relevance of identified		Checklist	Yes	
	Reasons for exclusion	State that you will provide a list of articles excluded at full text with		Checklist	Yes	
Critical appraisal	Critical appraisal strategy	Describe here the method you propose for critical appraisal of study	Optional	Checklist	n/a	
	Critical appraisal used in synthesis	Describe how the information from critical appraisal will be used in	Optional	Checklist	n/a	
	Consistency checking	Describe how repeatability of critical appraisal of study validity will be	Optional	Checklist	n/a	
Data extraction	Meta-data extraction and coding	Describe the method for meta-data extraction and coding for studies		Checklist	Yes	
Data synthesis and	Narrative synthesis strategy	Describe methods to be used for narratively synthesising the evidence	Vote-counting (tallying of studies based on the direction or significance	Checklist	Yes	
	Knowledge gap and cluster	Describe the methods to be used to identify and/or prioritise key		Checklist	Yes	
	Demonstrating procedural	Describe the role of systematic reviewers (who have also authored	Reviewers who have authored articles to be considered within the review	Checklist	Yes	
Declarations	Competing interests	Describe of any financial or non-financial competing interests that the		Checklist	Yes	

References

[1] James, K.L., Randall, N.P. and Haddaway, N.R., 2016. A methodology for systematic mapping in environmental sciences. Environmental Evidence, 5(1), p.7.

[2] Bayliss, H.R., Haddaway, N.R., Eales, J., Frampton, G.K. and James, K.L., 2016. Updating and amending systematic reviews and systematic maps in environmental management. Environmental Evidence, 5(1), p.20.

[3] Haddaway, N.R., Kohl, C., da Silva, N.R., Schiemann, J., Spök, A., Stewart, R., Sweet, J.B. and Wilhelm, R., 2017. A framework for stakeholder engagement during systematic reviews and maps in environmental management. *Environmental Evidence* , *6* (1), p.11.

[4] Collaboration for Environmental Evidence. 2018. Guidelines and Standards for Evidence synthesis in Environmental Management. Version 5.0. www.environmentalevidence.org/information-for-authors.

[5] Leeds Institute of Health Sciences. https://medhealth.leeds.ac.uk/info/639/information_specialists/1500/search_concept_tools. Accessed 12/11/2017.
